# High Dynamic Range Image Reconstruction from Saturated Images of Metallic Objects

**DOI:** 10.3390/jimaging10040092

**Published:** 2024-04-15

**Authors:** Shoji Tominaga, Takahiko Horiuchi

**Affiliations:** 1 Department of Computer Science, Norwegian University of Science and Technology, 2815 Gjøvik, Norway; 2 Department of Business and Informatics, Nagano University, Ueda 386-0032, Japan; 3 Graduate School of Engineering, Chiba University, Chiba 263-8522, Japan; horiuchi@faculty.chiba-u.jp

**Keywords:** material appearance, high dynamic range image reconstruction, metallic objects, reconstruction of saturated gloss, saturated low dynamic range images, deep neural network approach, HDR image database, gloss perception, human psychological experiments, LDR-to-HDR mapping

## Abstract

This study considers a method for reconstructing a high dynamic range (HDR) original image from a single saturated low dynamic range (LDR) image of metallic objects. A deep neural network approach was adopted for the direct mapping of an 8-bit LDR image to HDR. An HDR image database was first constructed using a large number of various metallic objects with different shapes. Each captured HDR image was clipped to create a set of 8-bit LDR images. All pairs of HDR and LDR images were used to train and test the network. Subsequently, a convolutional neural network (CNN) was designed in the form of a deep U-Net-like architecture. The network consisted of an encoder, a decoder, and a skip connection to maintain high image resolution. The CNN algorithm was constructed using the learning functions in MATLAB. The entire network consisted of 32 layers and 85,900 learnable parameters. The performance of the proposed method was examined in experiments using a test image set. The proposed method was also compared with other methods and confirmed to be significantly superior in terms of reconstruction accuracy, histogram fitting, and psychological evaluation.

## 1. Introduction

The appearance of a material can be a signature of quality and a criterion for object choice. Terms such as glossiness, matteness, transparency, metallic feel, and roughness are commonly used to describe the perceptual attributes of material appearance. This information not only helps in appreciating the beauty in life but also guides us in determining value. In recent years, the appearance of materials has become a crucial research topic in academia and industry [1]. The acquisition, modeling, and reproduction of material appearances are primarily based on two-dimensional images obtained by digital cameras.

Digital cameras can only capture a limited range of luminance levels in real-world scenes because of sensor constraints. High-quality cameras for high dynamic range (HDR) imaging are sometimes unaffordable. However, most existing image content has a low dynamic range (LDR), and most legacy content predominantly comprises 8-bit LDR images. Objects in real scenes do not always have matte surfaces and often have surfaces with strong gloss or specular highlights. In such cases, the pixel values in the captured images are saturated and clipped because of the limited dynamic range of image sensors, leading to missing physical information in saturated image regions.

**Figure 1:**
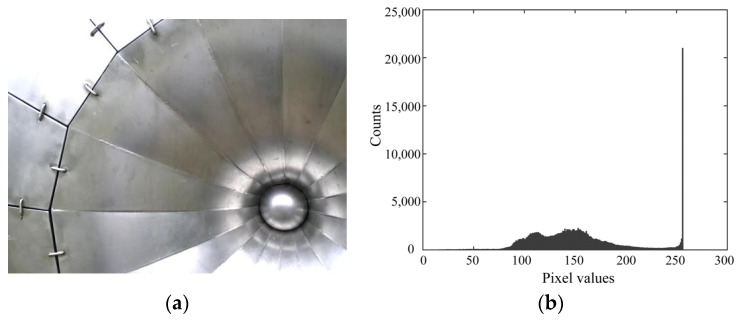
Example from the metal category in the Flickr material database image set: (**a**) color image named “metal_moderate_005_new”; (**b**) luminance histogram of the image in the 8-bit range.

Metals are typical object materials that saturate easily, and the luminance of the reflected light from a metal object covers an extensive dynamic range, all the way from matte surface reflection to specular reflection. Figure 1 shows an example from the Flickr material database ([2,3]), where the database is divided into 10 material categories: metal, plastic, fabric, foliage, and so on. All of these categories consist of 8-bit images. Figure 1a shows the color image named metal_moderate_005_new. Figure 1b shows the corresponding luminance histogram in the 8-bit range. A wide area of the metal object surface is saturated. The color and shading information in the saturated image area are entirely incorrect, and physical details are missing. Consequently, appearance modeling methods that attempt to reproduce appearance, such as gloss perception, fail for this object.

Therefore, a method is required to infer the original HDR image from a single LDR image suffering from saturation, often referred to as the inverse tone-mapping problem [4]. This is an ill-posed problem because a missing signal that does not appear in a given LDR image must be restored [5]. To date, this problem has been mainly addressed in the field of computer graphics [6,7,8,9,10,11] and partly in computer vision [5,12]. The target images are natural scenes and not material objects. Therefore, in addition to objects, the captured images contain the sky and various light sources.

This study targets the reconstruction of saturated gloss on an object surface. The HDR reconstruction of saturated gloss is important not only from a physical perspective, but also from a human psychology evaluation perspective. Studies on gloss perception are also underway [13,14,15], involving a complex interaction of variables, including illumination, surface properties, and observer. In recent years, neural networks have been applied to elucidate gloss perception [16,17], and the reconstruction of saturated gloss on object surfaces is a challenging research problem.

In this study, we consider a method for reconstructing the original HDR image from a single LDR image suffering from the saturation of metallic objects. A deep neural network approach is adopted to directly map the 8-bit LDR image to an HDR image. Note that there is no publicly available HDR image dataset; however, a few LDR datasets such as the Flickr material database are widely used. A small HDR image dataset in the preliminary work is shown in [18]. Therefore, we first construct an HDR image database specializing in metallic objects. A large number of various metallic objects with different shapes are collected for this purpose. These objects are photographed in a general lighting environment so that a strong gloss or specular reflection will be observed. Each captured HDR image is clipped to create a set of 8-bit LDR images. The pairs of created LDR images and original HDR images in the database are used to train and test the network.

We propose an LDR-to-HDR mapping method to predict the information that has been lost in saturated areas of LDR images. A convolutional neural network (CNN) was designed in the form of a deep U-Net-like architecture. The network consisted of an encoder, a decoder, and a skip connection to maintain high image resolution. Although the CNN approach with skip connections is known in machine learning, the effectiveness of such an approach was not shown for HDR image reconstruction from LDR images in the field of material appearance. Here is the first attempt for metallic objects.

In experiments, the performance of the proposed method is compared with those of other methods, examining in detail the accuracy of the reconstructed HDR images, which demonstrate the superiority of the proposed method in numerical error and histogram reconstruction validations. In addition to physical accuracy, perceptual faithfulness is also demonstrated through human psychological experiments.

## 2. HDR Image Database for Metallic Objects

**Figure 2:**
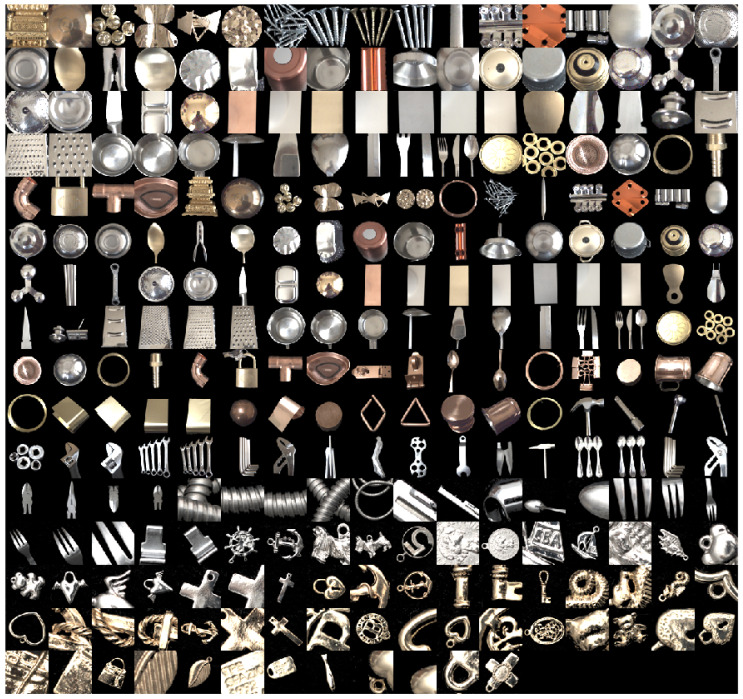
Set of material objects of different shapes and materials.

A large number of objects with different shapes made from different materials were collected. The collected material set consisted of a wide range of metallic materials, such as iron, copper, zinc, nickel, brass, aluminum, stainless steel, gold, silver, and metal plating. Painted metal objects were excluded from the analysis. The object shapes included not only flat plates but also various complicated curved surfaces. Figure 2 shows 267 metal objects collected in this manner. Light reflection from a metallic object consists of mostly specular reflection, rather than diffuse reflection [19]. The color appearing on the surface of an object is a metal color, coincident with a gloss/highlight color. We note that the color of the gloss/highlighted areas is not white. The metal colors are shown in Figure 2.

The metal objects were photographed using two types of cameras: an Apple iPhone 8 mobile phone camera with a depth of 12 bits and digital single lens reflex (DSLR) camera. For the details, including the spectral sensitivity functions, the reader is referred to [20]. The camera images were captured in the lossless Adobe digital negative (DNG) raw image format. A white reference standard was used for calibration. The DSLR camera was a Canon EOS 5D Mark IV, with a camera depth of 14 bits. Raw image data in CR2 format were converted into a 16-bit tiff to obtain images similar to the mobile phone camera format.

The lighting environment at the time of capture was based on a combination of three light sources: two fluorescent ceiling lamps and natural daylight through a window. During image capture, the surface of the metal object included glosses or highlights. Many images were captured by changing the shutter speed and lighting conditions for each metallic object in one-shot mode. Among the captured images, the image without saturation and with the highest dynamic range was used as the HDR image of the target object.

In place of a fixed lighting setup, a varying one was employed during the capture. Images were captured using the iPhone 8 camera under light from a fluorescent ceiling lamp and/or natural daylight from a window in a room, while the Canon EOS camera was used with a different type of fluorescent ceiling lamp in another room. These settings were not in laboratories but in actual rooms. The geometry and spectral power distributions of the three light sources varied, mitigating the risk of the learning process overfitting to a specific lighting environment.

Shading in a captured image is highly dependent on the positions of the object and camera. Therefore, by shifting the positions, multiple objects were photographed under different shading conditions. Thus, a set of 267 original images of metal objects was constructed, thereby erasing the backgrounds of target objects.

The original image was resampled to a size of 256 × 256 pixels. For data augmentation, each original image was geometrically varied by (1) image horizontal flipping, (2) zoom using the three factors of 1.0, 1.3, and 1.5, and (3) rotations using 13 angles of −90, −75, −60, −45, −30, −15, 0, 15, 30, 45, 60, 75, and 90 degrees. Accordingly, each original image had 78 modifications.

The processes for creating HDR and LDR are summarized as follows:

**(1) HDR creation**: The RGB pixel values of the acquired image were divided by the RGB values of the white reference, that is, the original image was normalized such that the RGB values of the white reference standard were set to 1 (8 bits). Subsequently, inverse gamma transformation was applied to compress the normalized images.

**(2) LDR creation**: The LDR images were created after clipping the HDR images and adjusting the final format to 8 bits following inverse gamma transformation.

The captured images had relative values based on the white reference standard. The white standard object of Minolta CR-A43 was placed near the target object and photographed along with the target object, and the camera values of the metallic object were normalized using the camera values of this white reference. If the luminance level of the object was the same as that of the white reference, the pixel value of *x* = 1.0.

**Figure 3:**
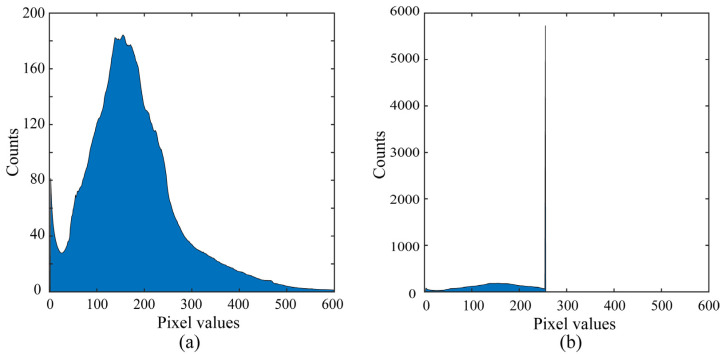
Average luminance histograms of the created image database: (**a**) average HDR image histogram; (**b**) average LDR image histogram suffering from saturation; images were clipped into the 8-bit range.

To compress the dynamic range for convenient data processing, the nonlinear transformation of inverse gamma correction was applied to pixel values *x*:

(1) $$y=x^{1/\gamma },$$

where *γ* was set to 2.0. Furthermore, the pixel values were converted using 255 × *y* to map the 8-bit LDR range to [0, 255]. Pixel values above this range were saturated in HDR. When the number of saturated pixels was small, we regarded this as noise. The saturated areas were assumed not to cover the entire object because in such a case, the saturated pixels cannot be recovered from a single LDR image. Based on these considerations, the ratio *R* of the saturated area to the total object area was calculated for each image. Subsequently, the saturated HDR image set satisfying the condition of 0.04≤R≤0.40 was adopted as being effective for the present study. The total number of HDR and LDR pairs in the image database created in this way was 14,535. Figure 3a displays the average luminance histogram for the HDR image database. The RGB pixel values covered a very wide range [0, 2010]. Figure 3b shows the average luminance histogram of the corresponding LDR image database suffering from saturation, with images clipped into the 8-bit range with a maximum of 255.

## 3. Method for Reconstructing HDR Image from Saturated LDR Image

### 3.1. Network Structure

**Figure 4:**
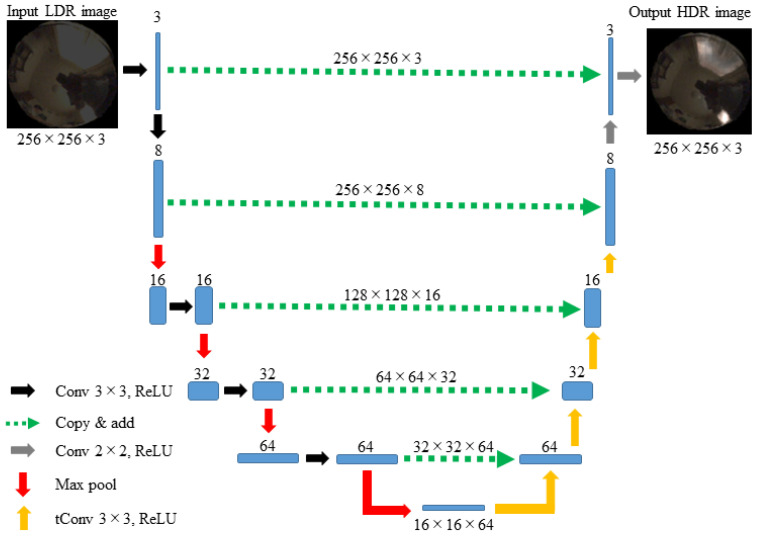
Entire network of this study. The abbreviations of **Conv**, **ReLU**, **Max pool**, and **tConv** represent convolution, rectified linear unit, max pooling, and transposed convolution, respectively. The green dotted arrows represent skip connections.

A deep-learning approach was considered to automatically predict a plausible HDR image from a single LDR image. Supervised learning was performed on the previously created image database using a deep CNN. The network designed in this study is shown in Figure 4 in its entirety. The network was designed as a U-Net-like architecture [21]. The LDR input image is transformed using the encoder network to produce a compact feature representation of the image, which is then input into an HDR decoder network to reconstruct the HDR image. The network is equipped with skip connections to maintain high image resolution.

The MATLAB machine learning functions were used to construct the designed networks [22]. An outline of the MATLAB code is presented in Appendix A. The entire network consisted of 32 layers, and the total number of learnable parameters was 85,900, which is the total number of coefficients of the weights and biases in the respective units.

### 3.2. Learning Procedure

Network training was performed using

**net = trainNetwork(ds_train, net_Layers, opts),**

where **ds_train** indicates the training dataset consisting of LDR and HDR pairs, and **opts** specifies several options, including the learning algorithm and learning rates. The loss function is defined as follows:

(2) $$E\left(\theta \right)=\frac{1}{2L}\sum_{i=1}^{K}{\left(t_{\;i}-y_{\;i}\left(\theta \right)\right)}^{2},$$

where the {$t_{\;i}$} are the pixel values of the target HDR image, and {$y_{i}$} are the reconstructed values predicted by the network. The vector **θ** is a large learnable parameter vector with 85,900-demensions; *K* is the total number of observations, *K* = 256 × 256 × 3, indicating the product of image size and RGB channels, and *L* is the mini-batch size, representing the number of samples used for training.

The stochastic gradient descent algorithm with a momentum term (SGDM) [23] was used for network training. The parameter vector $\theta_{t}$ at the *t*-th step is updated as follows:

(3) $$\theta_{i+1}=\theta_{t}-\alpha \nabla E\left(\theta_{t}\right)+\beta \left(\theta_{t}-\theta_{t-1}\right),\;$$

where the second and third terms on the right hand side of (3) represent the changing terms based on the gradient descent and momentum terms, respectively. The symbol $\nabla E\left(\theta_{t}\right)$ represents the gradient of loss function *E*, and the two scalars α and β represent the learning rate and contribution from the past, respectively.

In the present system, the training options are specified as follows:

Learning schedule: “piecewise”,

Initial learning rate of α: 2 × 10^−12^,

Learning rate drop factor for α: 0.96,

Drop period: 20,

Momentum β: 0.90,

Mini batch size *L*: 16.

The reconstructed HDR image predicted from an input LDR image was obtained using the trained network as follows:

**y = predict(net, ds_validation)*,***

where **net** is the network trained, and **ds_validation** indicates the LDR test dataset of images used for validation.

## 4. Experiments

### 4.1. Performances of the Proposed Method

The total number of HDR and LDR pairs in our image database was 14,535, of which randomly selected 12,000 were used for training the network, and the remaining 2535 were used as the validation data to investigate the proposed method. Each pair in the training data constituted the network input and output. One period of presenting the entire training dataset was defined as an epoch. The training was iterated for as many epochs as necessary to reduce the mean square error to an acceptable level. After 600 epochs, the root-mean-square error (RMSE) for the validation data was 16.95, where

(4) $$RMSE={\left\{\frac{1}{K}\sum_{i=1}^{K}{\left(t_{\;i}-y_{\;i}\left(\theta \right)\right)}^{2}\right\}}^{1/2}.$$

**Figure 5:**
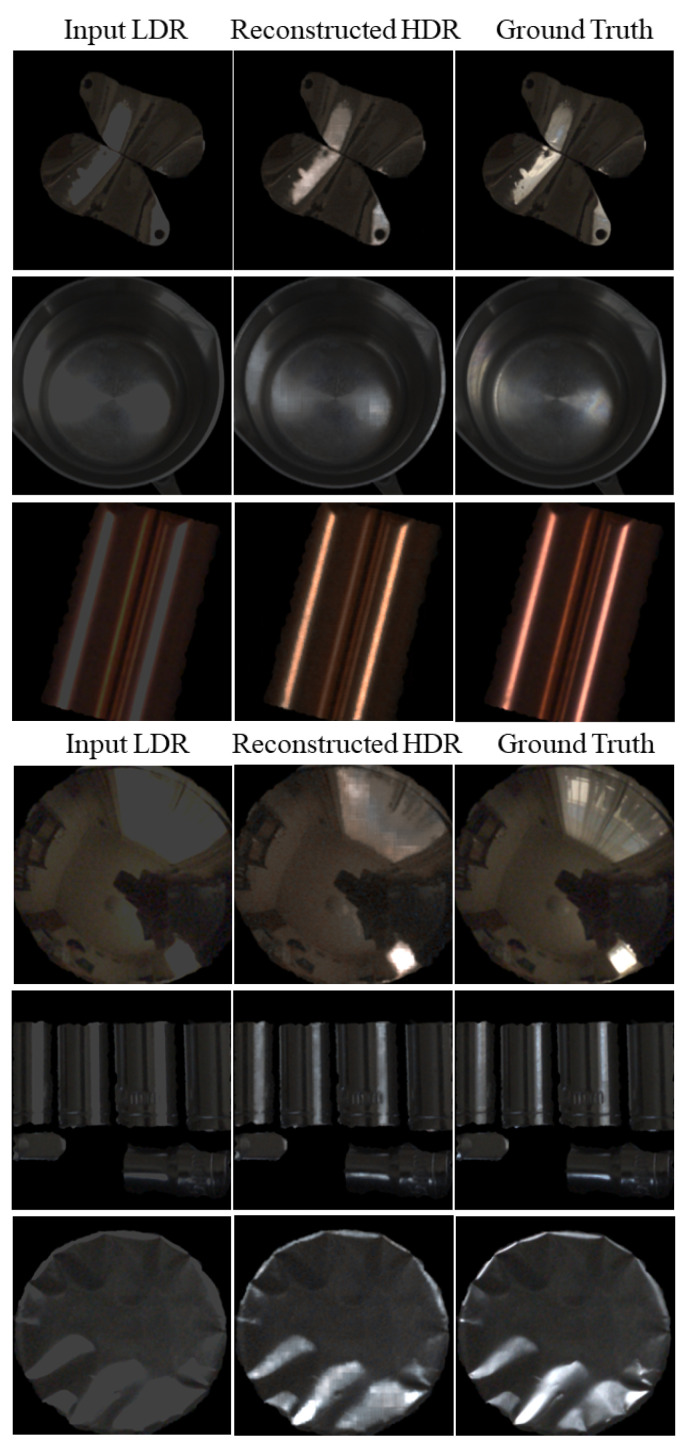
Input LDR image (**left**), reconstructed HDR image (**middle**), and original HDR image (**right**) compared for each metallic object.

Six samples were randomly selected from the validation image set to visually clarify the reconstruction results of the proposed method. Figure 5 compares the input LDR image (left), reconstructed HDR image (middle), and target HDR image (right) of the ground truth for each metallic object. Each image is displayed as a 16-bit tiff to prevent saturation. The LDR images were saturated in the 8-bit range; therefore, all LDR images were very dark, and the highlighted areas with saturation appeared gray.

Notably, not only the matte areas on the surface, but also gloss and highlighted areas have the same metallic object color. This characteristic differs from that of dielectric materials, such as plastic. For instance, gloss/highlighted areas on the reconstructed images for the third and fourth objects appear in the metallic colors of copper and gold. Thus, the HDR images reconstructed from the LDR images were well-recovered and close to the target HDR images.

**Figure 6:**
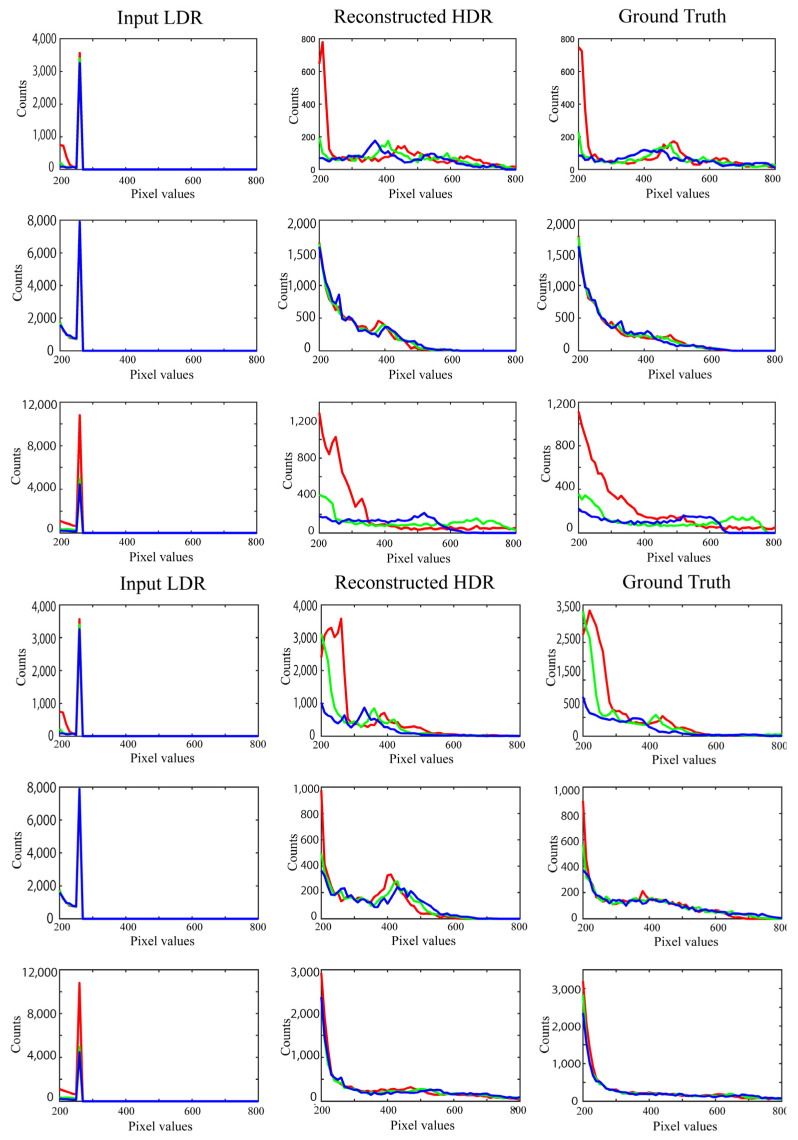
Comparisons of RGB histograms for each metallic object: LDR image (**left**), reconstructed HDR image (**middle**), and original HDR image (**right**). The respective RGB histograms correspond to the respective images shown in Figure 5. Red, green, and blue colors of lines correspond to RGB components.

In addition to validating the RMSE, we investigated the histogram distributions of RGB pixel values. Figure 6 depicts the histogram distributions, where the object images are the same as those shown in Figure 5. Note that if the pixel values in the reconstructed HDR image are not saturated, they are the same as those in the LDR image and the original HDR image without saturation. Therefore, we compared the histogram distributions in the saturated areas between the reconstructed and target images. Here, the histograms suffering from saturation are compared in the range [200, 800]. Figure 6 shows the RGB histograms of the LDR, reconstructed, and target images in the range of [200, 800], where the histograms correspond to the respective images shown in Figure 5. When the LDR histograms on the left are saturated at a pixel value of 255, they appear spiky, whereas the RGB histograms of the reconstructed HDR images are well recovered, approximating the histograms of the original HDR images.

**Figure 7:**
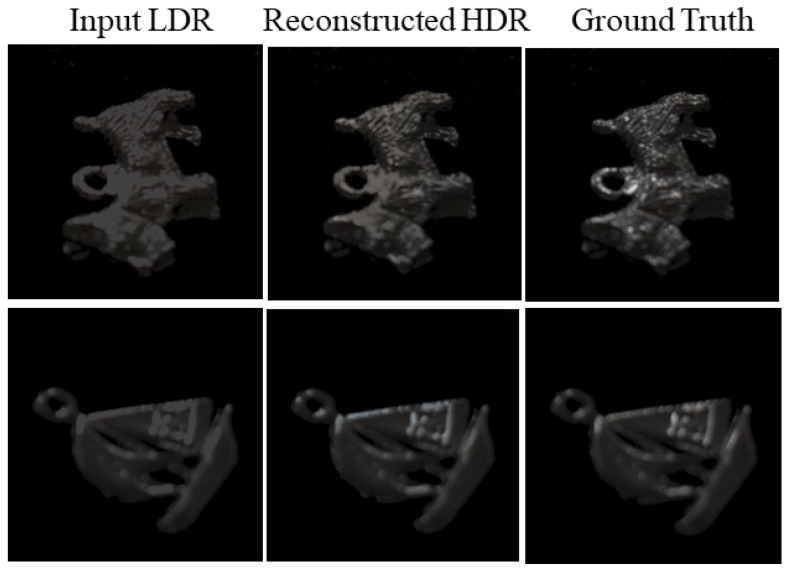
Input LDR image (**left**), reconstructed HDR image (**middle**), and original HDR image (**right**) compared for two new metallic objects.

Furthermore, we performed an additional experiment where the proposed method was applied to test the case not included in the database shown in Figure 2 nor the augmented dataset under entirely different lighting conditions. Figure 7 shows the results for two new metallic objects. The images were captured under two light sources; one is the fluorescent ceiling lamp and another is a LED light (FOSITAN L4500) placed diagonally upward at 45 degrees. The captured images suffered from saturation in the 8-bit range. Each LDR image suffering from saturation was input to the trained network in the previous section. Figure 7 compares the input LDR image (left), reconstructed HDR image (middle), and target HDR image (right) of the ground truth for the respective metallic objects. The average RMSE between the reconstructed HDR images and the target HDR images was 11.15, which was less than the RMSE for the validation data. The HDR images reconstructed from the LDR images are close to the target HDR images as seen in Figure 7.

**Figure 8:**
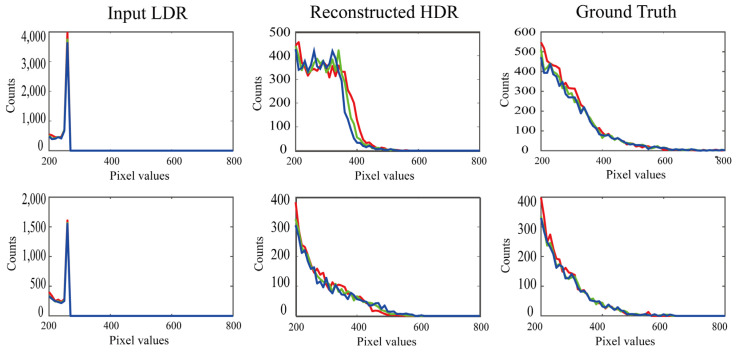
RGB histograms of the LDR, reconstructed, and target images in the range of [200, 800], where the histograms correspond to the respective images shown in Figure 7.

We also compared the histogram distributions in the saturated areas between the reconstructed and target images. Figure 8 shows the RGB histograms of the LDR, reconstructed, and target images in the range of [200, 800], where the histograms correspond to the respective images shown in Figure 7. The RGB histograms of the reconstructed HDR images are well recovered in the saturated range larger than 255, compared with the histogram of the LDR images.

### 4.2. Comparisons with Other Methods

#### 4.2.1. Numerical Performance

**Figure 9:**
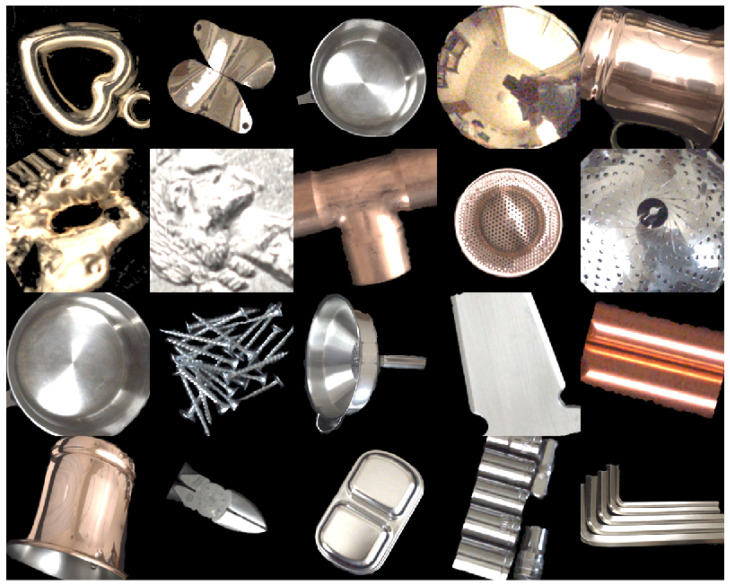
Test dataset of 20 object images used for the comparisons.

Another test dataset consisting of 20 images was randomly selected from the validation dataset. Figure 9 shows a set of HDR images for the 20 objects used for comparison with the other methods.

The following five algorithms that are open to the public were selected for performance comparisons. These methods were applied to reconstruct HDR images from LDR images of natural scenes and are not limited to metallic objects.

1. G. Eilertsen, et al. [8];

2. D. Marnerides, et al. [9];

3. Y.-L. Liu, et al. [12];

4. M. S. Santos, et al. [10];

5. B. Masia, et al. [6].

Methods 1–4 are based on a deep CNN approach, minimizing the mean squared error during HDR image reconstruction. Method 5 is based on a perceptual approach that is used in psychological studies with perceptual faithfulness being the quality criterion rather than physical accuracy.

**Figure 10:**
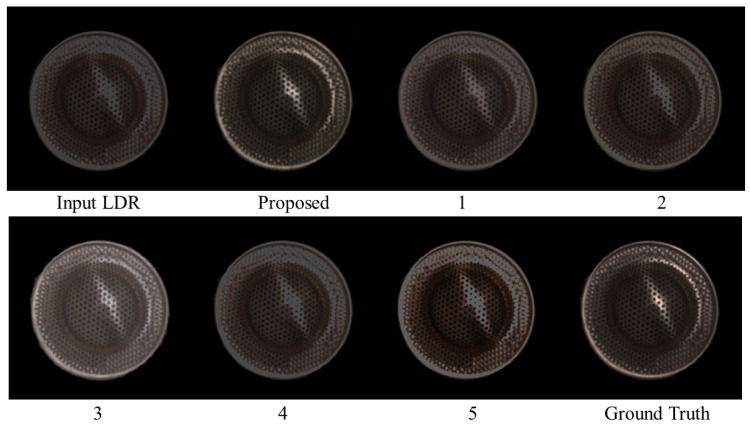
Comparison of the resulting images reconstructed by different methods for the ninth test sample.

**Figure 11:**
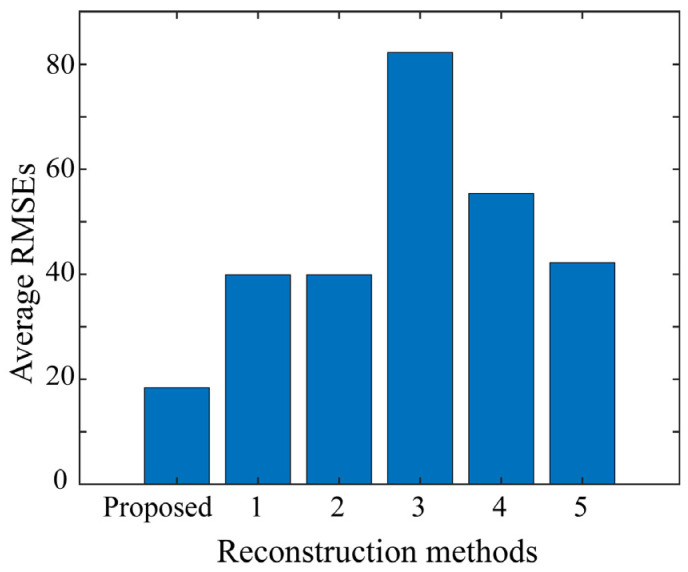
Average RMSEs over the entire test images used to compare the reconstruction accuracy numerically between six methods including the proposed method.

The respective algorithms were executed with saturated LDR images as input to reconstruct the HDR images. Figure 10 compares the resulting images reconstructed by the different methods for the ninth test sample. From left to right, the input LDR image, images restored using the proposed method, Methods 1 to 5, and the ground truth image (target HDR image) are arranged in order. The RMSE was calculated between the target and reconstructed images using each of the six methods, including the proposed method, to numerically compare reconstruction accuracy. Figure 11 presents the average RMSEs over all the test images in the bar graph. The proposed method has the lowest RMSE value, indicating its superiority in reconstruction accuracy compared to other methods.

#### 4.2.2. Histogram Reconstruction

**Figure 12:**
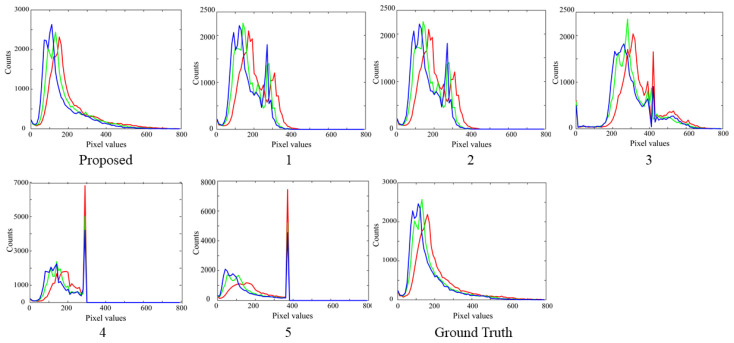
Comparison of the RGB histograms for the ninth test sample between the proposed method and Methods 1–5 in the range of [200, 800].

**Figure 13:**
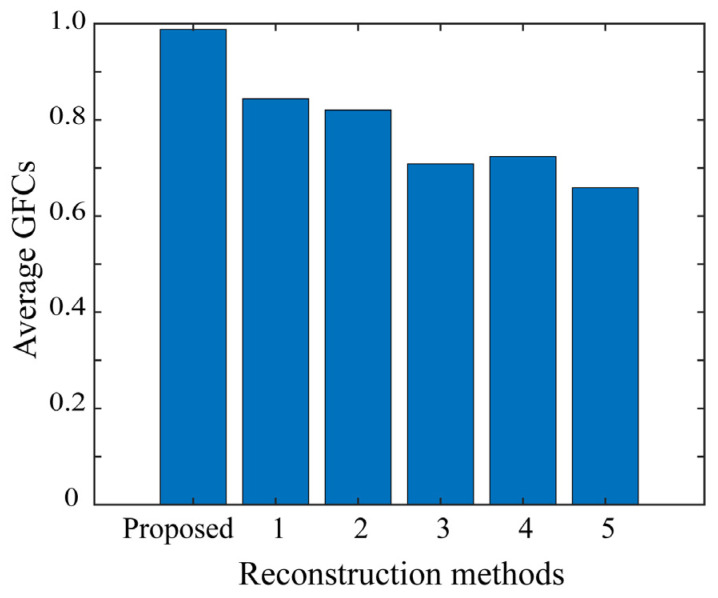
Average GFCs over the entire set of test images for the six methods including the proposed method.

Histogram reconstruction can be used to evaluate performance. Figure 12 compares the RGB histograms of the reconstructed images for the ninth test sample between the proposed method and Methods 1–5 in the range of [200, 800]. Compared with the other methods, the histograms of the proposed method are smooth and close to the ground truth. The goodness-of-fit coefficient (GFC) is useful for numerically evaluating histogram distributions [24]. This measure is the correlation coefficient between the predicted and target histogram curves. Let $h_{true}$ be a 61-dimensional column vector representing the histogram of the target HDR image in the range of [200, 800] in 10 steps, and let $h_{pred}$ be a 61 D column vector representing the histogram of the predicted HDR image in the same range. Then *GFC* is defined as

(5) $$GFC=\frac{h_{true}^{t}.*h_{pred}}{\left\|h_{true}\right\|\left\|h_{pred}\right\|},$$

where $h^{t}$ and $\left\|h\right\|$ indicate the matrix transposition and the norm of **h**, respectively, and the symbol (. *) represents elementwise multiplication. Figure 13 shows the average *GFC* values over all the test images in the bar graph. In the figure, there is no significant difference in the *GFC* values between the methods. A detailed inspection shows that the methods can be grouped into {1}, {2,3}, {3,4}, and {5}. Method 5 leaves the saturation part as is, simply performing gamma conversion. Therefore, histogram fitting does not perform well. The proposed method provides the highest *GFC*, close to the maximum value of 1, which means that the respective histograms reconstructed for the test samples fit very well with those of the target images, as shown in Figure 12.

#### 4.2.3. Human Psychological Evaluation

**Figure 14:**
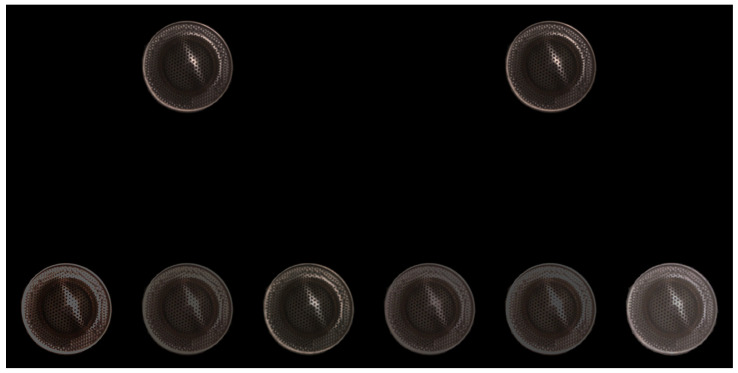
Example set of the HDR images displayed on the monitor for evaluation.

The psychological evaluation experiments were conducted using the dataset shown in Figure 9. Eleven observers participated in the evaluation experiments. The HDR images reconstructed by the proposed and other methods were displayed on a JAPANNEXT HDR monitor (JN-IPS2705UHDR) in a dark room. Figure 14 shows a sample set of the HDR images reconstructed for a target object displayed on the monitor for evaluation. The display screen included two reference images as the ground truth at the top and six test images at the bottom for evaluation, which were randomized using random numbers. The reproducibility of the six test images was evaluated relative to each other on a scale of 0–100. Detailed instructions for each observer to compare the HDR images are shown in Appendix B.

**Figure 15:**
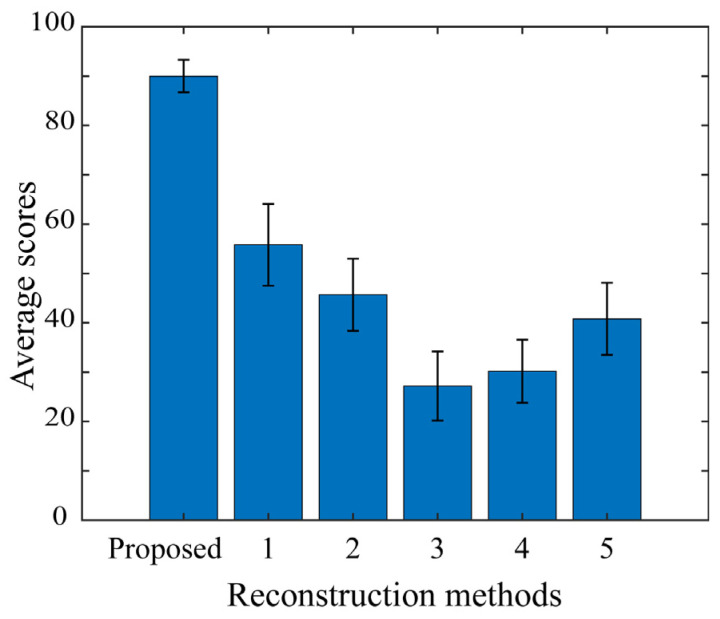
Average scores of the observer evaluation results for the reconstructed images over 20 target objects.

The bar graph in Figure 15 shows the average scores of the results when all 11 observers evaluated the sample images reconstructed for the 20 target objects twice. Each error bar represents the standard error of the mean score of each method for the 20 target objects. Note that this figure has the shape of the bar graph upside down displayed in Figure 11. Thus, it can be seen that the proposed method is clearly superior to all five of the other methods in psychological evaluations by human observers.

## 5. Conclusions

The reconstruction of saturated gloss on object surfaces is crucial for the acquisition, modeling, and reproduction of the appearance of a material. In this study, the proposed method reconstructs the original HDR image from a single saturated LDR image of metallic objects. First, an HDR image database was constructed from a large number of metallic objects with different shapes and of various materials. These objects were photographed using two different cameras in a general lighting environment to observe strong gloss or specular reflection. Each of the captured HDR images was clipped to create a set of 8-bit LDR images. The HDR and LDR images were represented by 256 × 256 pixels in each RGB channel. The total number of HDR and LDR pairs in the created image database was 14,535, split into training and testing sets.

Next, a method for reconstructing an HDR image from a single LDR image was proposed to predict the information lost in the saturated areas of the LDR images. A CNN approach was adopted to map the 8-bit LDR image directly to an HDR image. A deep CNN with a U-Net-like architecture was designed. The LDR input image was first transformed to produce a compact feature representation of the image, and then the HDR image was reconstructed. The network was equipped with skip connections to maintain a high image resolution. A network algorithm was constructed using MATLAB machine learning functions. The entire network consisted of 32 layers, and a total 85,900 of learnable parameters.

In experiments, we examined the performance of the proposed method using a set of test images for validation. The HDR images reconstructed from the LDR images were close to the target HDR images. The performance of the proposed method was validated based on RMSE values and RGB histogram distributions. The proposed method was also compared with other algorithms open to the public. The superiority of the proposed method was demonstrated not only in terms of quantitative accuracy based on RMSE and GFC, but also based on perceptual faithfulness in human psychological experiments.

The technical novelty of this paper lies in the combination of three aspects: the construction of an HDR image database for metallic objects, the development of a reconstruction method of HDR images from LDR images, and the evaluation of the performance for the HDR image reconstruction. Furthermore, our experimental findings indicate that the efficacy of converting LDR images to HDR images is unaffected by the material composition of metal objects but may be influenced by the objects’ shapes. For example, for a flat metal plate, depending on the lighting environment, the entire surface may have a strong specular reflection or the surface may become considerably dark. In other words, the reflections may change drastically depending on the lighting environment. In such cases, the performance of reconstructing the HDR image declines.

The proposed reconstruction method of the original HDR image from a single saturated LDR image is specialized to metallic objects. Among the numerous types of materials available, materials with strong gloss or specular reflection are limited to metals and dielectric materials such as plastic. The reflection of the metal was based only on specular reflection, whereas the reflection of the dielectric material was decomposed into two components: diffuse reflection and specular reflection. Therefore, saturated areas on dielectric objects often have the same color as the illumination. Addressing the challenge of reconstructing HDR images from saturated LDR images of dielectric objects remains a task for future research.

## Data Availability

Data are available on request from the authors.

## Appendix A. The Outline of the MATLAB Code

The network layers are constructed as follows:

**layers = [imageinputLayers, encodingLayers, decordingLayer].**

The encoding layers are described as:

**encodingLayers = **

**[convolution2dLayer(3, 8, …), reluLayer, maxPooling2dLayer(2, …, S’tride’, 2),**

**convolution2dLayer(3, 16, …), reluLayer, maxPooling2dLayer(2, …, S‘tride’, S),**

**convolution2dLayer(3, 32, …), reluLayer, maxPooling2dLayer(2, …, S‘tride’, S),**

**convolution2dLayer(3, 64, …), reluLayer, maxPooling2dLayer(2, …, S‘tride’, S)],**

where **convolution2dLayer(N, M, ...)** applies M sliding convolutional filters of size [N, N] to a 2D input image; the **reluLayer** performs a threshold operation on each element of the input, setting it to zero if the value is less than zero; and **maxPooling2dLayer(N, ..., S‘tride’, S)** performs down-sampling by dividing the input into rectangular pooling regions of size [N, N] and stride [S, S], thereby computing the maximum value of each region.

The basic decoding part is described as follows:

**decodingLayers = **

**[transposed_Conv2dLayer(2, 64, Stride=2), reluLayer,**

**transposed_Conv2dLayer(2, 32, Stride=2), reluLayer,**

**transposed_Conv2dLayer(2, 16, Stride=2), reluLayer,**

**transposed_Conv2dLayer(2, 8, Stride=2), reluLayer,**

**convolution2dLayer(1, 3,…), clippedReluLayer(1023.0), regressionLayer],**

where **transposed_Conv2dLayer(N, M, S‘tride’, S)** up-samples to a 2D feature map using M filters of size [N, N] and stride [S, S]; **clippedReluLayer(T)** performs clipping with upper limit T; and **regressionLayer** computes the half-mean squared error loss for the regression task.

## Appendix B. Description Given for the Experimental Procedure

Now, we explain the method used in the evaluation experiment. (When the lights are turned off, the display shows gray.) Please listen to the explanation while watching the display screen.

The experiment is for evaluating the reproducibility of the glossy areas of the metallic object in the image. At the start of the experiment, the display screen shows two reference images at the top and six test images at the bottom. The top two reference images are the same. You are asked to evaluate how well the glossy areas in each of the test images are adequately reproduced compared to the reference images. Adequately reproduced means that the overall brightness, color, and structure are similar in the reproduction.

You are asked to rate the reproducibility of the six test images relative to each other on a scale of 0 to 100. After determining the most and least reproducible among the six text images, the most reproducible test image is rated at 100 and the least reproducible as 0. For the remaining four images, the reproducibility rating varies linearly between 0 and 100. That is, if you determine the reproducibility to be between the highest and lowest, you rate the image as 50. Once the evaluation values are determined, you read them out loud, starting with the test image on the left. There will be a total of 20 sample images to evaluate. The same evaluation is repeated twice to increase reliability.

This concludes the description of the evaluation experiment. The collected data are handled under strict security to guard personal information and made public in a form that does not allow the individuals to be identified during the publication of the research results. You can take a break at any time during the experiment. If you feel sick during the experiment, you may interrupt or stop the experiment at any time, and do not hesitate to ask. Do you have any questions?
